# Mu Opioid Receptors on Primary Afferent Nav1.8 Neurons Contribute to Opiate-Induced Analgesia: Insight from Conditional Knockout Mice

**DOI:** 10.1371/journal.pone.0074706

**Published:** 2013-09-12

**Authors:** Raphaël Weibel, David Reiss, Laurie Karchewski, Olivier Gardon, Audrey Matifas, Dominique Filliol, Jérôme A. J. Becker, John N. Wood, Brigitte L. Kieffer, Claire Gaveriaux-Ruff

**Affiliations:** 1 IGBMC Institut de Génétique et de Biologie Moléculaire et Cellulaire, Translational Medicine and Neurogenetic Programme, UdS Université de Strasbourg, INSERM U964, CNRS UMR7104, Illkirch, France; 2 Molecular Nociception Group, Wolfson Institute for Biomedical research, University College London, London, United Kingdom; 3 ESBS, École Supérieure de Biotechnologie de Strasbourg, UdS Université de Strasbourg, Strasbourg, France; University of Sao Paulo, Brazil

## Abstract

Opiates are powerful drugs to treat severe pain, and act via mu opioid receptors distributed throughout the nervous system. Their clinical use is hampered by centrally-mediated adverse effects, including nausea or respiratory depression. Here we used a genetic approach to investigate the potential of peripheral mu opioid receptors as targets for pain treatment. We generated conditional knockout (cKO) mice in which mu opioid receptors are deleted specifically in primary afferent Nav1.8-positive neurons. Mutant animals were compared to controls for acute nociception, inflammatory pain, opiate-induced analgesia and constipation. There was a 76% decrease of mu receptor-positive neurons and a 60% reduction of mu-receptor mRNA in dorsal root ganglia of cKO mice. Mutant mice showed normal responses to heat, mechanical, visceral and chemical stimuli, as well as unchanged morphine antinociception and tolerance to antinociception in models of acute pain. Inflammatory pain developed similarly in cKO and controls mice after Complete Freund’s Adjuvant. In the inflammation model, however, opiate-induced (morphine, fentanyl and loperamide) analgesia was reduced in mutant mice as compared to controls, and abolished at low doses. Morphine-induced constipation remained intact in cKO mice. We therefore genetically demonstrate for the first time that mu opioid receptors partly mediate opiate analgesia at the level of Nav1.8-positive sensory neurons. In our study, this mechanism operates under conditions of inflammatory pain, but not nociception. Previous pharmacology suggests that peripheral opiates may be clinically useful, and our data further demonstrate that Nav1.8 neuron-associated mu opioid receptors are feasible targets to alleviate some forms of persistent pain.

## Introduction

Opiates such as morphine, acting at mu opioid receptors, represent the most widely used drugs for the management of severe pain. The three mu delta and kappa opioid receptors are major receptors for analgesia and are expressed at central and peripheral sites within the pain control circuits. Opioid receptors are also largely distributed in other neural pathways where they regulate reward and affective states [1-3]. Opioid receptors have been shown to inhibit pain transmission in ascending pain pathways including primary afferent fibers [4] synapsing second order neurons in the spinal cord as well as in the 'pain matrix' in the brain where pain messages are integrated [5]. Opioids also regulate descending inhibitory pain pathways by recruiting receptors in periacqueductal grey and rostral ventral medulla [5,6].

A major issue for pain alleviation by opiates is the manifestation of side effects including constipation, nausea as well as development of tolerance and addiction, often leading to arrest of treatments [7,8]. Furthermore, combined pharmacology and knockout approaches have confirmed that the molecular targets for the analgesic and side effects of the clinically used opiates are the mu receptors, encoded by the *Oprm1* gene [9]. Hence, drug treatments targeting opioid receptors outside the central nervous system are considered as a potential therapeutic strategy to limit centrally mediated side effects, and first trials using peripherally acting mu agonists at preclinical and clinical levels have been promising [4]. However, the significance of peripheral mu receptors in opiate-induced analgesia has not yet been investigated by gene knockout approaches.

In this study we examined the contribution of peripheral mu receptors to pain control using a conditional mouse knockout approach [10]. Primary nociceptive neurons comprise several cell populations [11] expressing Nav1.8 channels [12]. To delete mu opioid receptors in these sensory neurons, we first generated a mouse line harboring a floxed mu receptor gene. This floxed mu receptor mouse line was then crossed with a well-characterized mouse line expressing Cre recombinase in primary afferent Nav1.8 neurons including unmyelinated C, thinly myelinated Aδ, low-threshold mechanoreceptors and some Aβ fibers [13,14]. Recently, by using a similar approach, we have identified delta opioid receptors expressed by these same primary afferent neurons as major contributors to delta-agonist induced analgesia [15]. The present study shows that deletion of mu receptors in peripheral Nav1.8 sensory neurons decreases mu opiates-induced analgesia in inflammatory pain situation but not in acute nociception assays.

## Materials and Methods

### Ethics statement

All experiments were carried out in accordance with the European Communities Council Directive of 22 September 2010 (directive 2010/63/UE), with the guidelines of the Committee for Research and Ethical issues of IASP published in PAIN, 1983; 16:109-110, and were approved by the local ethical committee (Com’Eth, Comité d’Ethique pour l’Expérimentation Animale IGBMC-ICS, license N° 2010-003).

### Animals

The animals were bred and maintained in IGBMC. They were kept at 21-23°C and were provided with standard mouse chow and water ad libitum under a 12h light-dark cycle. Experiments were performed on male and female mice aged between 8 to 16 weeks. All behavioral testing was performed with the observer blind to the genotype or treatment.

### *Oprm1* floxed mice

**Figure 1:**
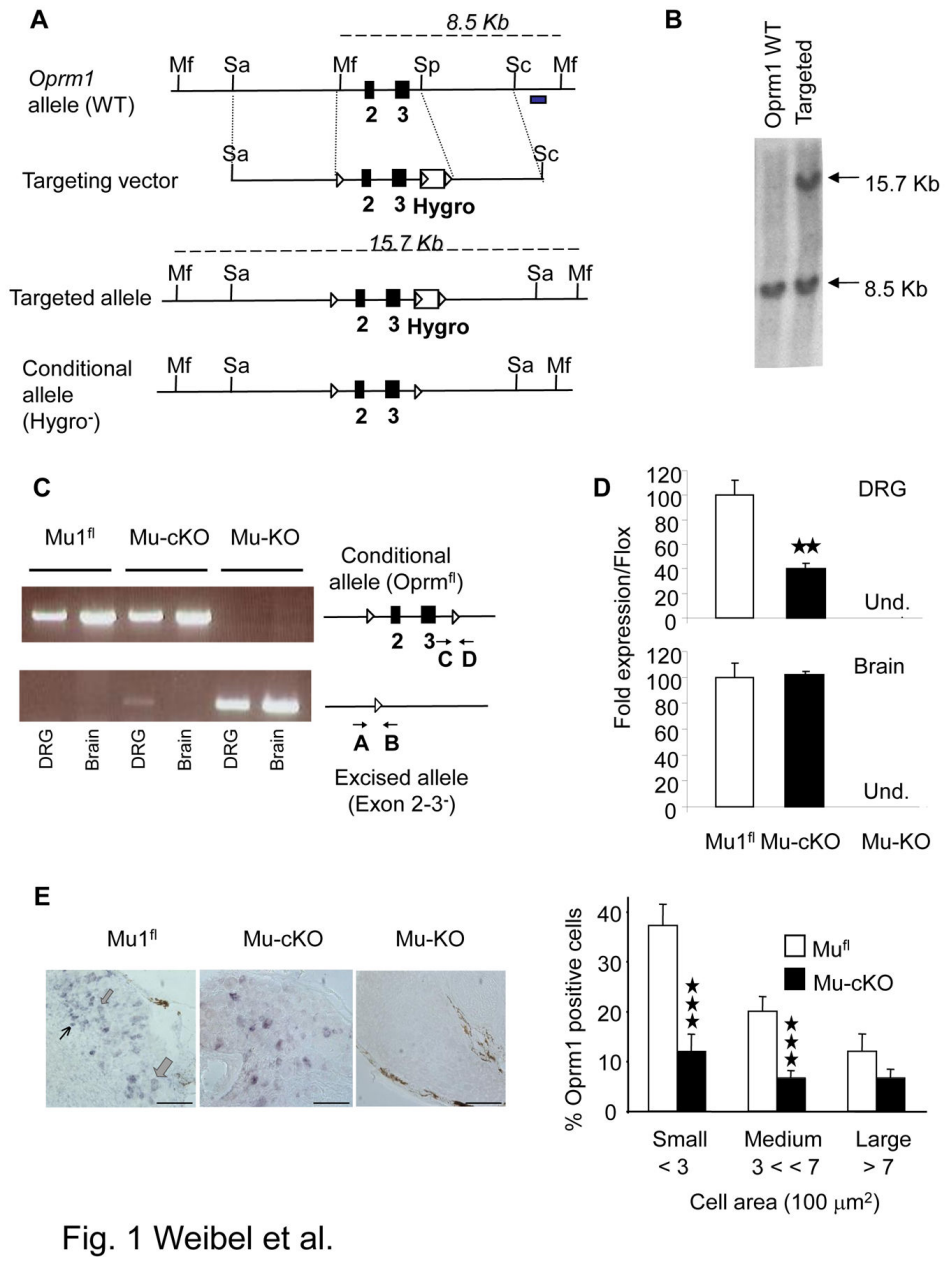
Generation of mu opioid receptor conditional knockout mice. (**A**) The conditional *Oprm1* (*Oprm1* ^fl^) allele was created by homologous recombination. The scheme shows the wild-type *Oprm1* allele, the targeting vector, targeted allele and conditional allele obtained after excision of Hygro^r^ by a Cre recombinase treatment of ES cells. The *Oprm1* ^fl^ conditional allele - or “floxed” allele - harbors two *loxP* sites flanking the *Oprm1* exon 2 and 3. Black boxes, exons; Mf, Mfe1; Sa, SalI, Sp, Spe1 restriction sites; white triangles, *loxP* sites; Hygro box, floxed hygromycin-resistance cassette, grey box, probe for Southern blot analysis. Dash lines indicate expected labeled DNA fragments in Southern blot analysis. (**B**) Southern blot analysis of wild-type and targeted alleles in ES cells. Genomic DNA was digested using Mfe1 and hybridized to a 3’ external probe, shown in 1A. The expected bands at 8.5 and 15.7 kb were obtained. (**C**) Conditional mutant mice. Right part shows the *Oprm1* ^fl^ conditional allele and excised allele (deletion of exons 2 & 3) after intercrossing *Oprm1* ^fl/fl^ mice with Nav1.8-Cre mice. A and B indicate PCR primers used to detect gene excision, and C & D PCR primers for the floxed allele. PCR shows exon 2-3 deletion in DRGs but not brain of mu-cKO mice. In DRGs, the two bands result from gene excision in Nav1.8^+^ neurons but not in other Nav1.8-negative cells. Mu-KO mice show full deletion in both DRGs and brain. (**D**) Conditional knockout of mu opioid receptor gene in DRGs but not brain. Quantitative RT-PCR was used to measure *Oprm1* mRNA levels from mu^fl^, mu-cKO and mu-KO mice. *Oprm1* mRNA expression was normalized to mu^fl^ control samples, and is decreased in mu-cKO animals. Oprm1 transcripts were undetectable in DRG and brain from mu-KO animals. ★★ *P*<0.01, mu-cKO *vs* mu^fl^ controls. (**E**) Conditional KO of the mu opioid receptor gene occurs in small/medium DRG cells. Left, representative *in* *situ* hybridization on DRG sections from mu^fl^, mu-cKO and mu-KO mice. Thin, medium and large arrows point to small, medium and large cells, respectively. Scale bar = 100 µm. Right, cell size distribution of *Oprm1*-positive neurons in DRGs. The % of *Oprm1*-positive neurons in control and mu-cKO DRGs are shown in white and black, respectively. The % of *Oprm1*-positive cells is significantly reduced in small and medium, but not large diameter (>700 µm^2^) neurons from mu-cKO mice. ★★★ *P*<0.001 mu-cKO *vs* mu^fl^ controls, Student t-test.

We generated mice with a floxed mu opioid receptor gene (*Oprm1* ^fl/fl^) where exon 2 and 3 are flanked by a *loxP* site (upstream) and a floxed hygromycin-resistance cassette (downstream) (see Figure 1A). A 6.8 kb genomic clone (SalI/SpeI) containing exons 2 and 3 of *Oprm1* gene was isolated from 129Sv genomic DNA and cloned into pBluescript plasmid to generate the targeting vector. This clone was engineered to introduce a *loxP* site 600 bp upstream of exon 2, and sequenced to verify *loxP* sequence. In order to increase the homologous regions surrounding the targeted locus, the construct was extended in the 3’ direction with a 3.5 kb SpeI-SacI fragment isolated from another mu receptor genomic clone containing a region downstream of exon 3. A vector containing the floxed hygro cassette was obtained from D. Metzger (IGBMC, Illkirch, France). The hygro cassette was removed from the cloning vector using NotI and SalI, and the fragment filled in using the Klenow fragment to generate blunt ends. Similarly, the targeting construct was digested with SpeI, blunted and the floxed hygro cassette ligated into the open site. The final 12.5 kb construct were checked by restriction and sequence analysis before being linearized for electroporation into 129Sv derived embryonic stem (ES) cells, which were selected with hygromycin. Surviving cells were screened for homologous recombination by Southern blotting with MfeI digests and using a 3' external probe (Figure 1A,B). Positive cells were transfected with a Cre recombinase-expressing plasmid for removal of the floxed-Hygro cassette. ES cells with the correct genotype were injected into C57BL/6J blastocysts, and resulting chimeric males were bred with C57BL/6J females to obtain germline transmission. F1 heterozygous *Oprm1* ^fl/+^ mice were intercrossed to generate the homozygous *Oprm1* ^fl/fl^ mouse line (50% C57BL/6J-50% 129Sv genetic background). We verified that *Oprm1* ^fl/fl^ mice show intact mu receptor expression by using a [^3^H]-DAMGO radioligand binding and a mu opioid agonist-stimulated [^35^S]-GTPγS binding assays on brain membrane preparations [15]. The mu opioid ligand [^3^H]-DAMGO bound similarly to brain membrane preparation from wild type and *Oprm1* ^fl/fl^ mice, as shown by comparable Kd (1.53 ± 0.18 nM wild-type; 2.04 ± 0.31 nM *Oprm1* ^fl/fl^, respectively) and Bmax (108 ± 11 pmol/mg protein wild-type; 109 ± 6 pmol/mg protein *Oprm1* ^fl/fl^, respectively) values. DAMGO induced a similar dose-dependent increase of [^35^S]-GTPγS binding in preparations from the two genotypes (EC50 315± 31 and 331 ± 44 nM; E_max_ 178 ± 7% and 175 ± 5% of basal activity, respectively), indicating that insertion of *LoxP* sites did not disrupt gene transcription. *Oprm1* ^fl/fl^ mice were maintained for 5 generations on the 50% C57BL/6J-129Sv genetic background until crossing with Nav1.8-Cre mice.

### Conditional Nav1.8-Oprm1^-/-^ knockout (mu-cKO) mice

In order to delete mu receptors specifically from peripheral nociceptive neurons, we crossed the *Oprm1* ^fl/fl^ mice with Nav1.8-Cre mice (100% C57BL/6J genetic background) that express Cre recombinase in Nav1.8 positive neurons [13]. F1 mice (75% C57BL/6J-25% 129Sv) were backcrossed to *Oprm1* ^fl/fl^ mice (50% C57BL/6J-50% 129Sv) to generate F2 mice (62.5% C57BL/6J-37.5% 129Sv). F2 mice homozygous for floxed *Oprm1* allele and heterozygous for Nav1.8-Cre were selected and further bred to produce experimental cohorts containing 50% conditional knockout animals (Nav1.8-*Oprm1* ^-/-^, referred as to mu-cKO throughout the study) and 50% littermate control animals (referred as to mu^fl^ throughout the study) that were on the same 62.5% C57BL/6J-37.5% 129Sv genetic background. As genetic background represents an important factor influencing pain assays [16], the conditional knockout mice (mu-cKO) mice and Cre-negative controls (mu^fl^) used in the study were littermates harboring the same genetic background. Genotyping of animals was done using PCR from tail genomic DNA for Nav1.8-Cre allele as previously described [15].

### Global CMV-*Oprm1^-/-^* knockout (mu-KO) mice

To evaluate whether *LoxP* sites in *Oprm1* ^fl/fl^ mice were functional *in vivo*, we bred *Oprm1* ^fl/fl^ mice (50% C57BL/6J -50% 129Sv) with CMV-Cre mice (100% C57BL/6J) that express Cre recombinase under the cytomegalovirus promoter [15], leading to germline deletion of *Oprm1* exon-2 and 3. These mice, referred as to mu-KO, were on a 75% C57BL/6J -25% 129Sv background and used as controls for molecular studies. Mu-KO animals showed a complete deletion of exon 2 and 3 in genomic DNA from both brain and DRGs (Figure 1C). Mu opioid agonist-induced G protein signaling ([^35^S]-GTPγS binding assay) was undetectable in these full-KO mice (not shown). Conventional *Oprm1* knockout animals [17] were also used in the tail immersion, hot plate, and capsaicin assays to verify the selectivity of morphine for the mu receptor in these tests.

### Analysis of *Oprm1* gene inactivation in mu-cKO mice

We used PCR on genomic DNA to test for *Oprm1* exon 2 and 3 deletion in DRG and brain from mu-cKO mice by using the *Oprm1* forward primers A (5’-ACCAGTACATGGACTGGATGTGCC-3’) and C (5’-GTTACTGGAGAATCCAGGCCAAGCC-3’) and reverse primers B (5’-TGCTAGAACCTGCGGAGCCACA-3’) and D (5’-CGCTTGGGAATATCTTGTACCTATGACCA-3’) for the excised and intact bands, respectively.

### In situ mRNA hybridization

Percentages of neurons expressing *Oprm1* transcripts in DRGs from mu^fl^ and mu-cKO mice were determined by *in situ* mRNA hybridization. DRG were isolated and snap-frozen in dry ice. In situ hybridization using non-radioactive Dig-dUTP labeled antisense and sense *Oprm1* probes as control were performed on cryostat sections of DRG (14 µm) as described previously [15]. The *Oprm1* probe (608 bp) encompassed most of exon 2 and 3 sequences. Pictures from epifluorescence microscope were taken using a coolSNAP camera, and image analysis was done using ImageJ. The number of positive cells was determined on representative transverse sections of DRGs. Mean diameters were determined for neurons showing a cross-sectioned nucleus. Per genotype, 1200 cells from 2 animals were analyzed from naïve animals (Figure 1E). For inflamed animals, 850 cells from ipsilateral DRGs per each genotype from 2 animals were analyzed.

### Quantitative RT-PCR

Quantitative RT-PCR was performed as described [15] on brains or pooled DRG from individual mice. Briefly, total RNA was extracted using TRIzol (Invitrogen, Cergy Pontoise, France). RNA were evaluated using a ND-1000 Nanodrop spectrophotometer and gel eletrophoresis. Total RNA (1 µg) from each DRG pool was reverse-transcribed in a final volume of 20 µl. Real-time PCR was performed on cDNA in triplicate on a Light-Cycler-480 instrument (Roche). Primer sequences were GACGGCCAGGTCATCACTAT (*β-actin* forward), CCACCGATCCACACAGAGTA (*β-actin* reverse), 5’-GAGCCACAGCCTGTGCCCT-3’ (*Oprm1* forward), 5’-CGTGCTAGTGGCTAAGGCATC-3’ (*Oprm1* reverse). Relative expression ratios (mu-cKO vs mu^fl^) were calculated by using actin as reference gene and the 2-ΔΔCt method to evaluate differential expression levels.

### Mu opioid agonist-stimulated [^35^S]-GTPγS binding assay on spinal cord membrane

Spinal cord membranes were prepared from mu-cKO, mu-KO and mu^fl^ control mice as described [18]. Samples were incubated in triplicate with and without the mu opioid receptor agonist DAMGO (10^-9^ to 10^-4^ M), for 1 hour at 25°C in assay buffer containing 30 µM GDP and 0.1nM [^35^S] GTPγS (NEG030H PerkinElmer). Non-specific binding was defined as binding in the presence of 10 µM GTPγS, and basal binding indicates binding in the absence of agonist.

### Nociception

Nociception assays were performed as described [15,19]. Briefly, the tail immersion test was performed by immersing the tail (5 cm from the tip) into a water bath at 48°C, 50°C and 54°C for the measurement of heat nociceptive responses. Tail withdrawal latencies were determined, with a cut-off of 10 s. Nociception to cold was measured by a tail immersion test in a water bath maintained at 5°C with a cut-off time of 40 s. The time taken by the mouse to withdraw its tail or whole body reaction was recorded. For the analysis of tolerance to morphine-induced antinociception, tail immersion was measured at 52°C with a 10 s cut-off. For determining morphine-induced antihyperalgesia in the CFA-tail model, tail immersion was measured at 48°C with a 20 s cut-off.

Tail flick test was conducted by exposing the tail of mice to radiant heat, using an analgesymeter (LE7306 Panlab, Bioseb, France). Tail withdrawal latencies were determined, and a cut-off of 20 s was set.

For the Hargreaves plantar test, the radiant heat source (Bioseb, France) was focused on the plantar surface of the hind paw. The time from initiation of radiant heat until paw withdrawal was measured automatically (withdrawal latency in sec.), with a cut-off of 20 s.

The hot plate test was performed by placing mice on the hot plate (Bioseb, France) set at 48°C, 50°C and 54°C for the measurement of heat nociceptive responses. Latency for the first sign of hind paw discomfort (hind paw licking, shaking or jumping) was recorded with a 120 s cut-off. Morphine-induced antinociception was determined with the hot-plate set at 54°C with a 120 s cut-off.

Mechanical nociception was conducted by applying a gradual increasing pressure by the pressure stimulation unit with conic tip (Randall-Selitto apparatus, Bioseb, France). Pressure threshold of tail withdrawal were determined, with a 560 g cut-off value.

Mechanical sensitivity was determined by probing the plantar surface of hind paws with von Frey filaments, according to the up-and-down method. Plantar hind paw surface was stimulated with a series of eight von Frey filaments (bending force ranging from 0.008 to 2 g). The threshold of response was calculated by using the up-down Excel program provided by the Alan Basbaum’s Laboratory (UCSF, San Francisco, USA).

Acetic acid-induced visceral response (writhing) was induced and measured as described [20] by injecting intraperitoneally (ip) 0.1ml/10g body weight of 0.6% acetic acid in water. Acetic acid injection produced a reaction characterized by contractions of the abdominal musculature followed by extension of the hind limbs. Mice were placed in individual transparent cages and the number of writhes par mouse during the 10 min period between 5 and 15 min after acetic acid injection was counted.

The capsaicin test was performed as described [19]. Briefly, 1.6 µg capsaicin in 10 µl were injected subcutaneously (s.c.) into the dorsal surface of the right mouse hind-paw. The behavioral manifestations of nociception (paw licking and flinching) were measured during the 10 min after capsaicin injection.

### Inflammatory pain

Complete Freund’s adjuvant (CFA, 8 µl) was applied to one hind-paw to induce inflammatory pain, as described [15]. Mechanical allodynia was measured by using von Frey filaments and the up-down method [21], and heat hyperalgesia was assessed with the Hargreaves plantar test [15,22]. Spontaneous pain behavior was evaluated by scoring guarding behavior of CFA-injected hind paw as described [23] with modifications. Mice were placed in clear Plexiglas boxes (7x9x9 cm) on a mesh screen and scoring began when exploration and grooming behavior ended (15-30 min). Guarding behavior (paw elevation time) was assessed during 3 consecutive 2 min observation periods with 30 min intervals, and the sum of elevation time over the total 6 minutes was calculated. Inflammatory pain was also induced by injecting 20 µl into tail and assessing heat hypersensitivity by tail immersion at 48°C as described [24].

### Morphine-induced constipation

Measures for morphine-induced constipation was adapted from previous studies [25,26]. Transit of the small intestine was measured using the charcoal test. Six hours before testing, animals were fasted with free access to water. Mice received an injection of morphine or saline 20 min prior to oral gavage with a charcoal meal containing an aqueous solution of 5% charcoal (C3345, Sigma-Aldrich) and 2.5% arabic acid (A3006, Sigma-Aldrich). After 30 min, mice were euthanized by cervical dislocation and the small intestine dissected out. The distance travelled by the leading edge of charcoal was measured relative to the total length of the small intestine. The % of gastrointestinal transit was calculated as (charcoal distance/small intestine length) X 100. For analysis of fecal boli accumulation, mice were provided water and food ad libitum prior to the test. Mice were injected s.c. with morphine or saline and individually placed in an empty cage with a wired mesh. Fecal boli were collected on the paper beneath and weighed 4 h after injection.

### Administration of opiates

Morphine chlorhydrate (Francopia, Gentilly, France) in saline or saline control solution were injected ip for tail immersion, tail flick and tail pressure tests and subcutaneously (sc) for the acetic acid writhing tests, and animals tested 30 min later. Morphine or saline was injected ip 45 min before the hot plate test. In inflammatory pain experiments, morphine, fentanyl (F-3886, Sigma-Aldrich) and loperamide (L-4762, Sigma-Aldrich) were assayed 48 hours post-CFA, when inflammatory pain was well established. Morphine was injected ip for evaluation of systemic activity and mice tested 45 minutes later in the Hargreaves and then Von Frey filaments assays. Fentanyl (Sigma-Aldrich, France) was injected sc for assessment of systemic activity, and mice tested 15 min later. To test for naloxone methiodide (NM, N-129, Sigma-Aldrich) under CFA pain, the opioid antagonist was injected ip 20 min before sc morphine and analgesia was investigated 45 min after morphine injection. For testing local administration of NM, 48 hours post-CFA naloxone methiodide was administered intraplantarly (i.pl. 5 µg) into the CFA-injected hind paw in a 5 µl volume followed 1 min later by sc loperamide and test for analgesia 20 minutes later.

### Chronic morphine protocol for tolerance to antinociception

The induction and measure of tolerance to analgesia were performed as described [27]. Mice were injected ip for 4 days twice a day (9 a.m. and 6 p.m.) with escalating doses morphine chlorhydrate (10 to 40 mg/kg) in saline or saline control solution. Heat nociceptive thresholds were measured by using the tail immersion assay at 52°C. On day 5, antinociceptive response was tested with morphine cumulative dose-responses. Mice were given ascending doses of morphine (5, 10, 20 and 50 mg/kg s.c.) every 30 min and the tail withdrawal latencies were assessed. The cut-off value was set at 10 s.

### Statistical analysis

All data are presented as means ± sem. Comparison between genotypes for acute nociception was analyzed with Student’s *t* test. For inflammatory pain, comparison between genotypes was performed using repeated measures ANOVA followed by Fisher *post-hoc* test for individual time points when appropriate. The analysis of opiates effects was performed using two-way ANOVA for genotype and treatment followed by *post-hoc* Fisher’s test to determine statistically significant differences.

## Results

### Mice with a specific deletion of mu receptors in primary afferent Nav1.8^+^ neurons

We used the Cre-Lox strategy to delete mu opioid receptors (encoded by the *Oprm1* gene) specifically in peripheral Nav1.8^+^ sensory neurons. We first generated a mouse line with *Oprm1* ^fl^ conditional allele - or “floxed” allele - harboring two *loxP* sites around *Oprm1* exon 2 and 3 (Figure 1A-1B). We verified that insertion of *loxP* sites into the *Oprm1* gene did not modify mu receptor expression (see Material and Methods Section). To evaluate whether *LoxP* sites in *Oprm1* gene are functional *in vivo*, we bred *Oprm1* ^fl/fl^ mice with CMV-Cre transgenic mice that express Cre recombinase throughout the mouse body. CMV-Cre-*Oprm1* ^-^ (or mu-KO) animals showed a complete deletion of exon 2 and 3 genomic DNA in both brain and dorsal root ganglia (DRGs) (Figure 1C), indicating that the *loxP* sites in *Oprm1* gene were functional to produce Cre-mediated gene deletion. In order to delete mu receptors specifically from peripheral sensory neurons, we then crossed *Oprm1* ^fl/fl^ mice with mice expressing Cre recombinase under the Nav1.8 sodium channel promoter control. This driver Nav1.8-Cre line has been successfully used to produce the conditional inactivation of several genes in primary afferent Nav1.8^+^ neurons [13,28]. We analyzed genomic DNA from homozygous floxed animals harboring the Nav1.8-Cre transgene (mu-cKO mice). PCR analysis showed successful excision of *Oprm1* exons 2 and 3 in DRGs (Figure 1C). The floxed allele was intact in brain of mu-cKO animals (Figure 1C).

We next compared *Oprm1* gene expression in DRGs from mu-cKO and mu^fl^ control mice. Quantitative RT-PCR analysis showed a 60% decrease of *Oprm1* mRNA in DRGs from mu-cKO mice as compared to controls (Figure 1D, mu-cKO vs mu^fl^ *P* = 0.0019) while intact expression was found in the brain. *In situ* hybridization (ISH) revealed a large decrease in the number of *Oprm1*-expressing neurons in DRGs from mu-cKO mice (Figure 1E). DRGs from mu-cKO mice showed only 7% positive cells as compared to 29% *Oprm1*-expressing neurons in control mice (mu-cKO vs mu^fl^ control, *P* < 0.001). The decrease in *Oprm1*-expressing neurons was detected in small and medium neurons with a cell body area < 500 µm^2^, and was not significant in larger neurons (Figure 1E, P= 0.18, mu-cKO vs mu^fl^ for neurons > 500 µm^2^), indicating that the conditional mu receptor knockout occurred essentially in small/medium size neurons.

**Figure 2:**
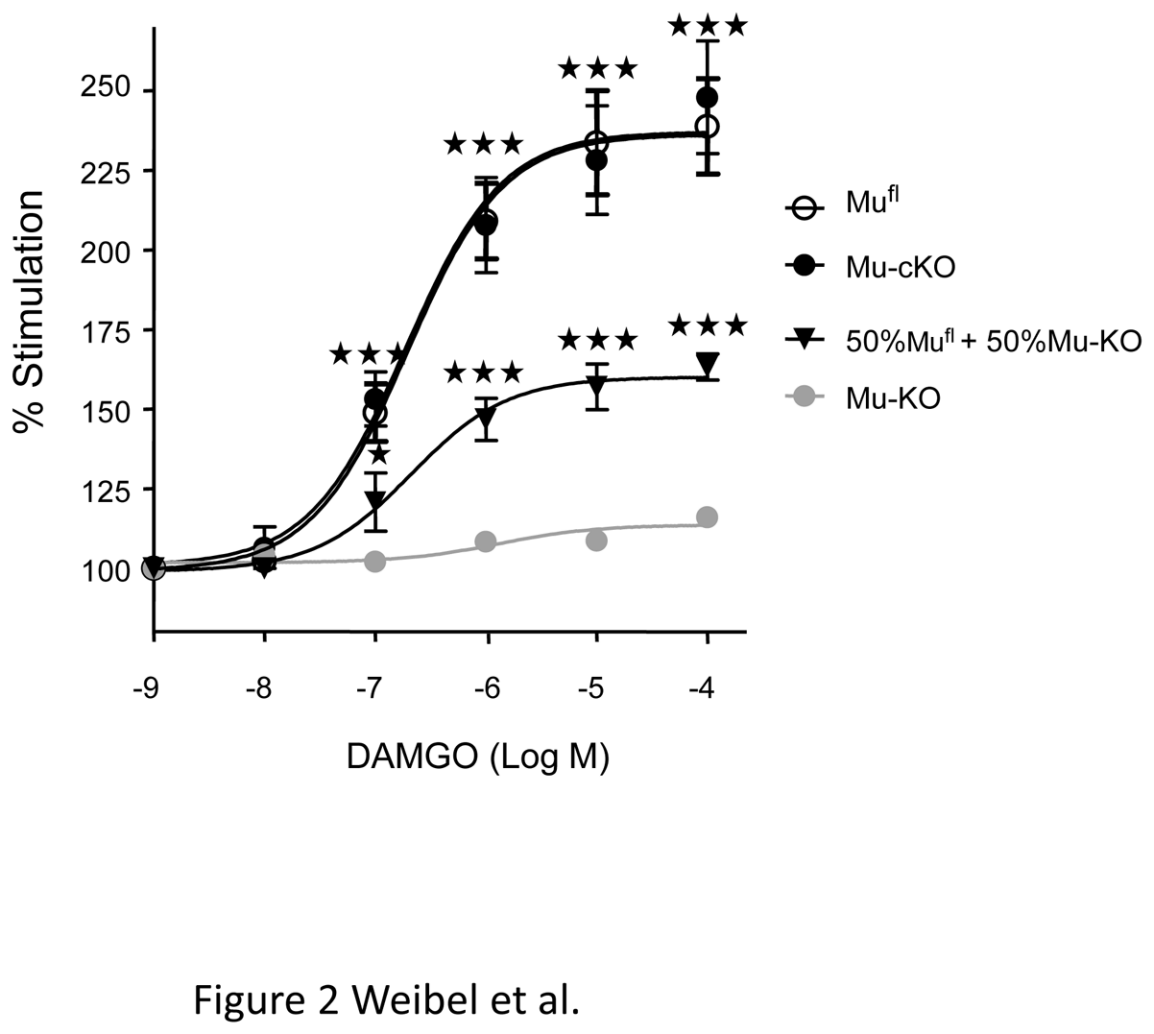
Mu opioid agonist-induced [^35^S]-GTPγS binding is comparable on spinal cord membrane preparation from mu-cKO and mu^fl^ mice. Spinal cord membranes were incubated in the absence or presence of the mu opioid agonist DAMGO (10^-9^-10^-4^ M) in assay buffer containing [^35^S] GTPγS. Basal level (100%) represents the specific [^35^S]-GTPγS binding in the absence of agonist. DAMGO significantly increases [^35^S]-GTPγS binding, in a comparable manner for mu-cKO and mu^fl^ mice. [^35^S]-GTPγS binding was absent on spinal cord membranes from mu-KO mice, and was decreased by half with a 50%-50% mix of membranes from mu^fl^ and mu-KO animals, containing half mu receptors as compared to mu^fl^ membranes, indicating that this assay allows to detect reduced receptor expression. Results are presented as means ± sem of 5-6 experiments on 5 distinct membrane preparations. ★★★ *P*<0.001, ★ *P*<0.05 50%-50% mu-cKO, mu^fl^ or 50%-50% mix *vs* mu-KO, Student t-test.

To determine if the deletion of mu receptors in primary Nav1.8 neurons would impact on mu receptor levels in the spinal cord, we compared mu receptor protein expression of mu-cKO and control mu^fl^ mice by using the mu opioid agonist DAMGO-stimulated [^35^S]-GTPγS binding assay on spinal cord membrane preparations. Mu opioid receptor activation levels were indistinguishable in spinal cords from mu-cKO and control animals (Figure 2). We verified that we could detect a decrease in mu receptor expression level by testing a 50%-50% mix of spinal cord membranes from mu^fl^ and mu-KO animals. As expected, mu receptor stimulation for this mix was half as compared to that obtained with spinal cord membrane from mu^fl^ animals. This finding suggests that conditional mu receptor knockout in Nav1.8 neurons did not induce a substantial reduction of mu receptor binding in the spinal cord.

### Nociceptive thresholds are similar in mu-cKO and control mice

We first examined the consequences of conditional mu receptor deletion on acute pain perception. As mu receptor activation is traditionally known to reduce the perception of heat pain, we first compared mu-cKO and control animals in the heat nociception paradigms that were used previously to characterize conventional receptor KO animals [17,20,29-31].

**Figure 3:**
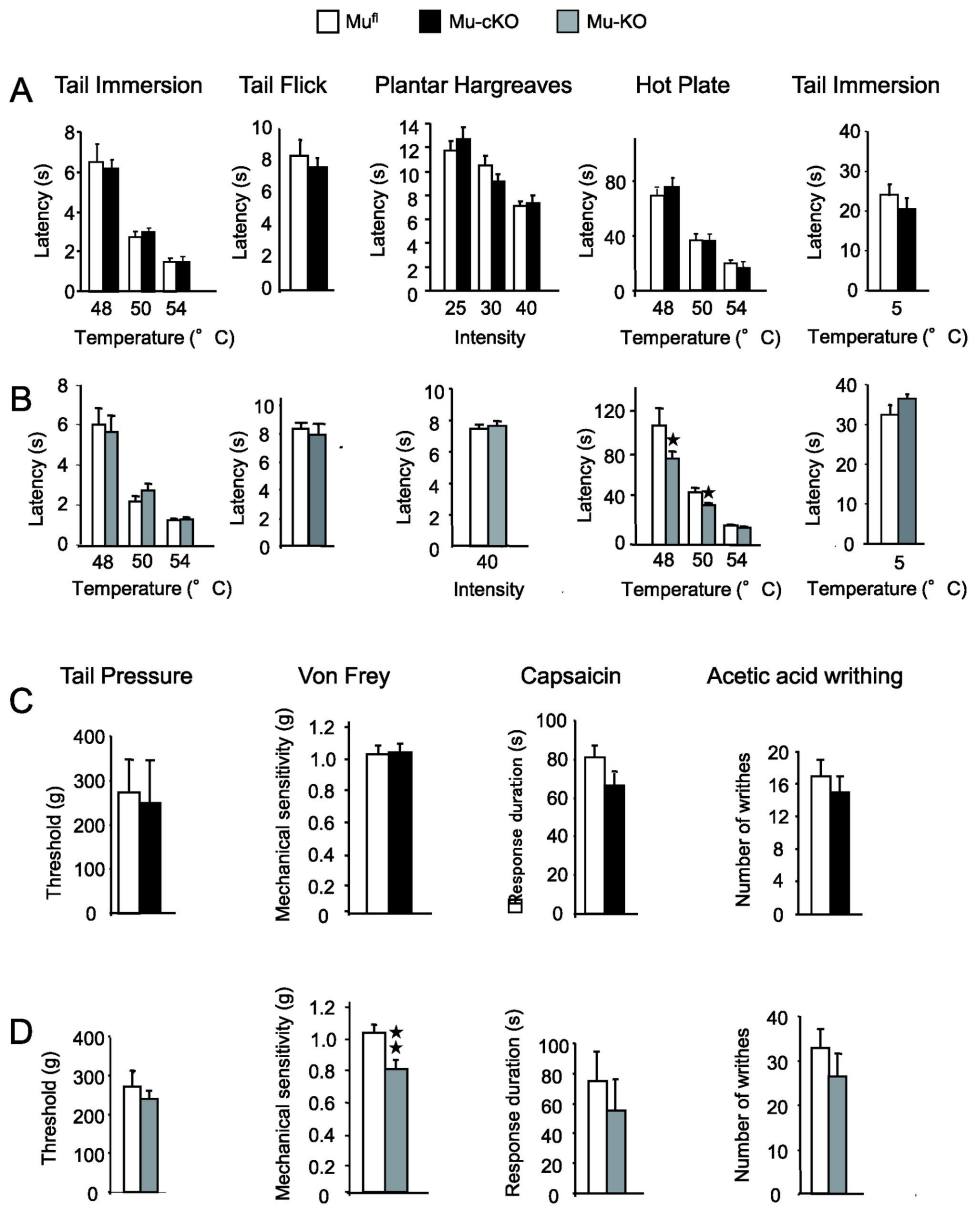
Acute pain responses are unchanged in conditional mu-cKO mice. (A) Acute thermal responses were similar in mu-cKO mice and mu^fl^ controls in the tail immersion test at 48, 50 and 54°C (n=15/genotype), tail flick test (n=10/genotype), Hargreaves test at three different intensities (n=10/genotype), hot plate test (48, 50 and 54°C, n=15 /genotype) and cold tail immersion test at 5°C (n=19/genotype). (B) In the experimental conditions of (A), mu receptor total knockout mice displayed higher sensitivity in the hot plate assay only (conventional KO vs WT, n =11-14/genotype, ★ *P*<0.05) whereas they behaved as controls for heat tail immersion (n=11-14/genotype), tail flick (n=12/genotype), Hargreaves (n=20/genotype) and cold (5°C) tail immersion (n=12-13/genotype) tests. (C) Nociceptive responses to mechanical and chemical stimuli were unchanged in the conditional mutant mice when assessed in the tail pressure (n = 8/genotype) or von Frey filaments (n = 10-13/genotype) test, nocifensive responses to capsaicin (n=10/genotype) and abdominal writhing induced by acetic acid (n=13-14/genotype). (D) In the same experimental conditions as in (C), mu receptor total knockout mice were more sensitive than control mice in the von Frey filaments test for touch perception (conventional KO vs WT, n = 33-57/genotype, ★★ *P*<0.01, Student t-test). Total knockout mice behaved as controls in the tail pressure test (n = 8/genotype), nocifensive responses to capsaicin (n=6/genotype) and abdominal writhing induced by acetic acid (n=4/genotype).

Conditional mu-cKO mice showed no alteration of responses to noxious heat in the tail immersion, tail flick, and hot plate assays (Figure 3A). Under the same conditions, mu-KO (or conventional-KO) mice displayed higher sensitivity in the hot plate assay (Figure 3B), as previously reported [29-31]. When cold nociception was investigated in a 5°C tail immersion assay, mu-cKO, mu-KO and mu^fl^ animals responded with similar tail withdrawal latencies (Figure 3A right panels). Also, no change was found in cKO animals for response to mechanical stimuli in von Frey filaments and tail pressure assays, as well as responses to chemical stimuli in the capsaicin and acetic acid-induced visceral pain assays (Figure 3C). Under similar conditions, total mu-KO mice showed enhanced mechanical sensitivity in the Von Frey test (Figure 3D). Altogether, our results indicate that the endogenous tone at mu receptors expressed by primary afferent Nav1.8 neurons does not control acute pain perception to heat, mechanical and chemical stimuli.

### Morphine-induced antinociception and tolerance to antinociception are unaltered in cKO mice

**Figure 4:**
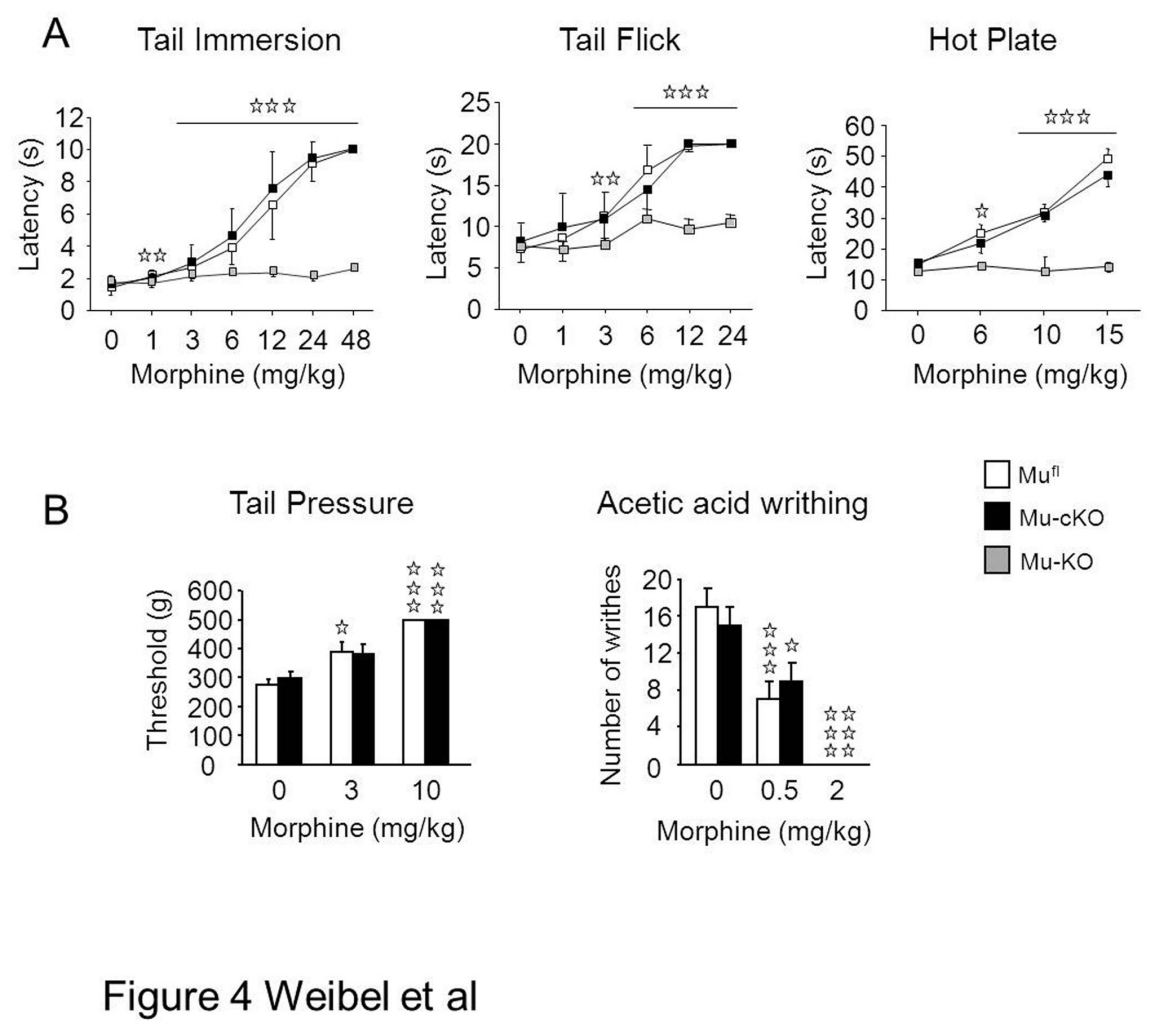
Conditional mu-cKO and control mice show comparable systemic morphine analgesia in nociceptive assays. Top. Morphine induced dose-dependent antinociception in both mu-cKO and mu^fl^ mice in the three heat assays, (**A**) tail immersion, (**B**) tail flick and (**C**) hot plate. Morphine-induced analgesia was abolished in conventional mu-KO animals (tail immersion, n=10/genotype; tail flick, n=10-14/genotype; hot plate, n=6-17/ genotype), confirming the selective effect of morphine on mu receptor. Bottom. Mu-cKO and mu^fl^ control mice show similar systemic morphine analgesia in the tail pressure (n=9-13/genotype) and acetic acid-induced visceral nociceptive (n=13-14/genotype) assays. Two-way ANOVA, post-hoc Fisher test for individual time points, ✰ *P* <0.05, ✰**✰ *P*<0.01**, ✰✰✰ *P*<0.001, morphine *vs* saline.

We examined morphine-induced antinociception in several acute pain responses well known to be morphine-sensitive and widely used for opiates analgesia [9]. As the tail flick, tail immersion and hot plate heat pain assays may implicate peripheral receptors to a different extend, we have investigated the role of the targeted mu receptors in these three assays. Morphine produced dose-dependent antinociception in the tail flick, tail immersion and hot plate assays, which were comparable in mu-cKO and mu^fl^ mice (Figure 4A). *In vivo* morphine selectivity for mu receptor at the tested doses was verified in conventional knockout animals (Figure 4A). Morphine induced also similar analgesic responses in mu-cKO and control animals for tail pressure and visceral acetic acid writhing assays (Figure 4B). Globally, this indicates that mu receptors expressed by Nav1.8 sensory neurons do not contribute to systemic morphine-induced antinociception under acute noxious conditions.

**Figure 5:**
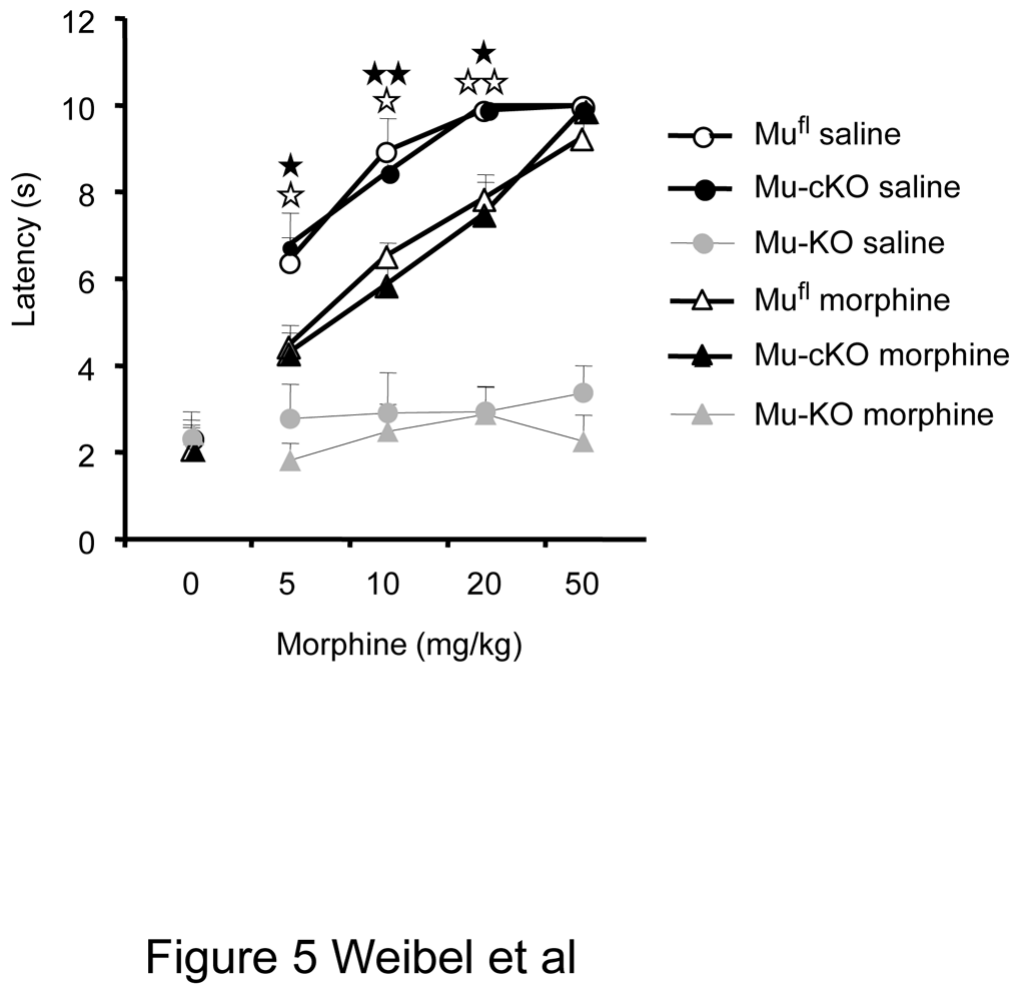
The conditional deletion of mu receptor in Nav1.8 primary neurons does not abrogate tolerance to morphine-induced antinociception. Morphine dose-dependent antinociception was measured following repeated 4-day i.p. injections of morphine or saline in mu^fl^ controls, mu-cKO and mu-KO animals. The shift to right for both mu^fl^ and mu-cKO chronic-morphine animals indicates the development of a comparable tolerance to analgesia. Total mu-KO animals show no antinociception. n=6-7/genotype/treatment, two-way ANOVA (genotype x treatment F(1,30) = 19.919, *P* <0.001 for treatment; F(2,30) = 97.039, *P* <0.001 for genotype); post-hoc Fisher test for individual morphine doses, ✰ *P* <0.05, ✰✰ *P*<0.01, chronic morphine (tolerance) *vs* chronic saline in mu^fl^ mice; ★ *P*<0.05, ★★ *P*<0.00 chronic morphine (tolerance) *vs* chronic saline in mu-cKO mice.

We also determined if the targeted mu receptors were implicated in the development of tolerance to antinociception (Figure 5). We treated mu^fl^, mu-cKO and mu-KO mice with repeated doses of morphine (10 to 40 mg/kg twice per day) over 4 days to induce tolerance, and investigated at day 5 morphine-induced antinociception by using the tail immersion assay at 52°C, as described in a recent study [27]. Control saline mu^fl^ and mu-cKO mice displayed the same dose-response curve for morphine-induced antinociception (morphine ED50 in mu^fl^ 5.02 ± 1.02 mg/kg; mu-cKO 5.40 ± 1.12 mg/kg; *P*> 0.05 between genotypes). Chronic morphine administration induced tolerance to antinociception, shifting morphine dose-response curve to the right in both genotypes (chronic morphine vs chronic saline, mu^fl^ *P*= 0.012; mu-cKO *P*= 0.03). There was no significant difference between chronic morphine-treated mu^fl^ and mu-cKO mice (morphine ED50 in mu^fl^ 10.01 ± 1.36 mg/kg; mu-cKO 13.09 ± 3.01 mg/kg; *P*> 0.05 between genotypes). Mice with the total deletion of mu receptor showed no antinociceptive response to morphine, as expected. Thus, mu receptors on Nav1.8 sensory neurons do not appear to be mainly implicated in tolerance to analgesia.

### Mu opiate-induced analgesia is decreased in cKO mice under inflammatory pain

**Figure 6:**
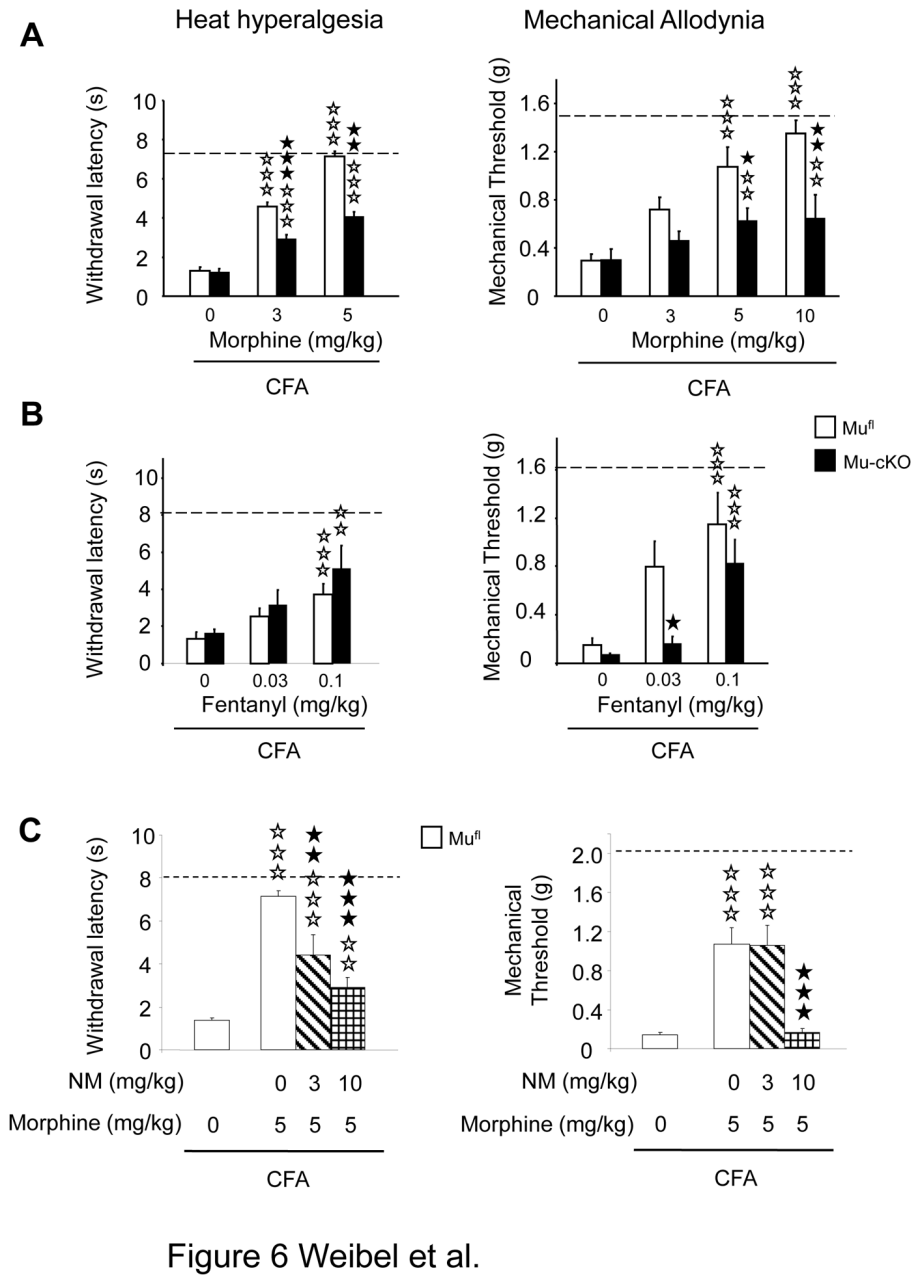
Conditional mu-cKO mice show decreased opiate-induced analgesia in the CFA-induced inflammatory pain model. (**A**) Two days after CFA, morphine (i.p.) dose-dependently reduced heat and mechanical hypersensitivities in mu^fl^ control mice. This analgesia was diminished in mu-cKO mice. Dashed lines represent baseline (pre-CFA) sensitivities. White bars, mu^fl^ mice; black bars, mu-cKO mice. For plantar test, n=13-20/genotype. two-way ANOVA (genotype x treatment F(1,90) = 75.336, *P* <0.001 for treatment; F(1,90) = 23.313, *P* <0.001 for genotype. Post-hoc Bonferroni test, ✰✰✰ *P*<0.001, morphine *vs* saline; ★★ *P*<0.01, ★★★ *P*<0.001 cKO vs flox controls. For Von Frey filaments test, n=7-18/genotype. two-way ANOVA (genotype x treatment F(1,91) = 41.573, *P* <0.001 for treatment; F(1,91) = 27.378, *P* <0.001 for genotype. Post-hoc Bonferroni test, ✰✰ *P*<0.01, ✰✰✰ *P*<0.001 morphine *vs* saline; ★ *P*<0.05, ★★ *P*<0.01 mu-cKO vs mu^fl^ controls. (**B**) Fentanyl produced a dose-dependent analgesia in mu^fl^ control mice 2 days after CFA. Mu-cKO mice displayed a decreased analgesic response in the mechanical sensitivity test for the 0.03 mg/kg fentanyl dose. n=5-10/genotype. For plantar test, two-way ANOVA (genotype X treatment F(1,54) = 8.979, *P* <0.001 for treatment, *P*= 0.19 for genotype), post-hoc Bonferroni test; ✰✰ *P* <0.01, ✰✰✰ *P*<0.001, fentanyl 0.1 mg/kg *vs* saline; for fentanyl 0.03 mg/kg, *P* = 0.0675 in mu^fl^ animals, *P* = 0.21 in mu-cKO animals. For Von Frey test, two-way ANOVA (genotype X treatment F(1,45) = 22.802, *P* <0.001 for treatment, F(1,45) = 9.316, *P* <0.01 for genotype). Post-hoc Bonferroni test for treatment, ✰✰✰ *P*<0.001, fentanyl 0.1 mg/kg *vs* saline; for fentanyl 0.03 mg/kg, *P* = 0.0575 mu^fl^ animals, *P* = 0.28 mu-cKO animals. Post-hoc Fisher test for genotype, ★ *P*<0.05, mu-cKO vs mu^fl^ mice. (**C**) Morphine-induced analgesia is reduced by systemic administration of the peripheral antagonist naloxone methiodide (NM). Morphine (5 mg/kg) induced an antihyperalgesic effect in mu^fl^ mice for both heat and mechanical responses. Systemic NM diminished morphine-induced analgesia. Heat hypersensitivity, one-way ANOVA for treatment F(3,42) = 29.778, P<0.001 ; post-hoc Bonferroni test, ✰✰ *P* <0.01, ✰✰✰ *P* <0.001 morphine *vs* saline; ★★ *P*<0.01 ★★★ *P*<0.001 NM + morphine *vs* morphine. Mechanical hypersensitivity, one-way ANOVA for treatment F(3,52) = 23.467, P<0.001 ; post-hoc Bonferroni test, ✰✰✰ *P* <0.001 morphine *vs* saline; ★★★ *P*<0.001 NM + morphine *vs* morphine.

**Figure 7:**
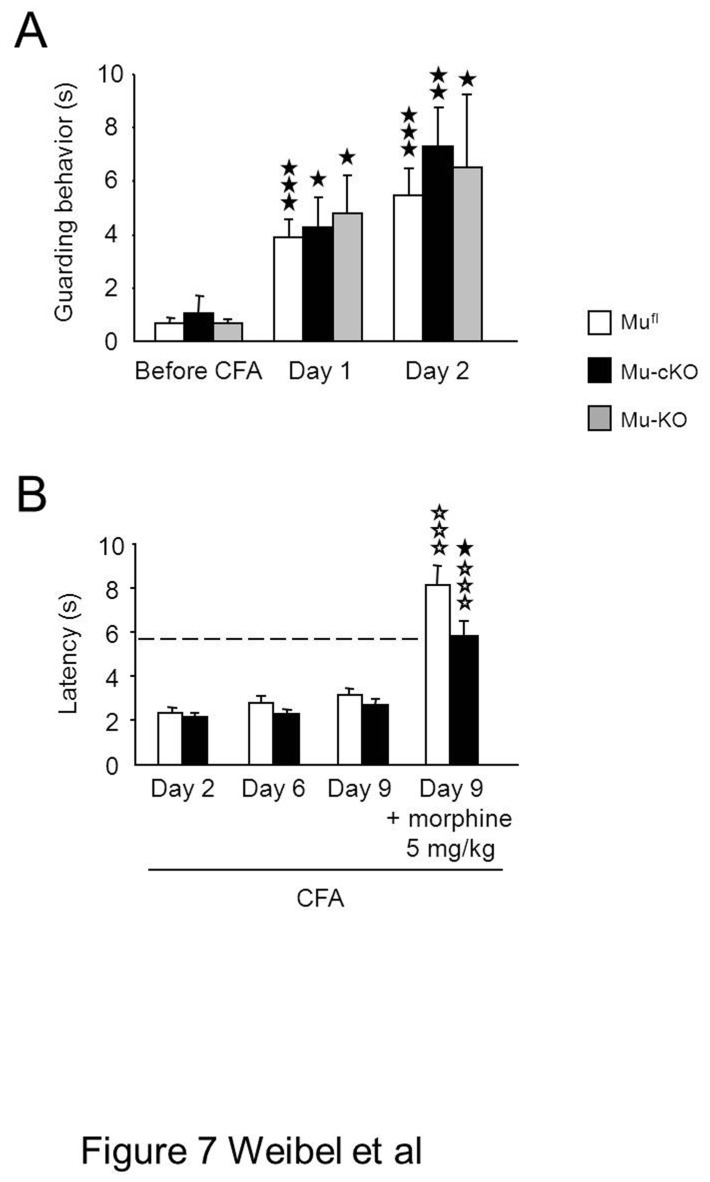
Spontaneous guarding pain behavior after paw-CFA and CFA-inflammatory pain at day 9 in conditional mu-cKO mice. (**A**) The effect the conditional mutation on ongoing pain behavior was evaluated by quantifying the duration of guarding behavior over 6 min in mu^fl^, mu-cKO and mu-KO mice before CFA-induced inflammation and at days 1 and 2 post-CFA. All mouse lines showed the same behavior (mu^fl^, n=14; mu-cKO, n=6; mu-KO, n=12). Results are expressed as means ± sem. ★ *P*<0.05, ★★ *P*<0.01, ★★★ *P*<0.001 post-CFA *vs* naïve. (B) Following CFA injection into tail, mu-cKO and mu^fl^ mice showed similar heat hyperalgesia at days 2, 6 and 9. The dashed line represents baseline (pre-CFA) sensitivity in the tail immersion tests at 48°C. Morphine (i.p.) produced anti-hyperalgesia in both genotypes, and that was reduced in mu-cKO mice as compared to controls. n=19/genotype., two-way ANOVA (genotype x treatment F(1,71) = 48.812, *P* <0.001 for treatment; F(1,71) = 5.999, *P* <0.05 for genotype. Post-hoc Bonferroni test, ✰✰✰ *P*<0.001, morphine *vs* saline; ★ *P*<0.05, cKO vs flox controls.

When inflammatory pain was induced locally by injection of Complete Freund Adjuvant (CFA) into a hind paw, mu-cKO and mu^fl^ control mice displayed comparable thermal and mechanical hypersensitivities 48 h post-CFA (Figure 6A and B; 0 mg/kg morphine). Also, there was no difference for spontaneous pain as measured by guarding behavior, under CFA-inflammation (Figure 7A). Therefore, our results indicate that the endogenous tone at mu receptors expressed by Nav1.8 neurons does not exert a tonic control on the hypersensitivity induced by CFA-inflammation.

Interestingly, the peripheral mu receptor KO modified opiate-induced analgesia under conditions of inflammatory pain. Morphine administered by a systemic route dose-dependently attenuated CFA-evoked hypersensitivity in control mu^fl^ animals. However morphine was less effective in the mu-cKO mice. Analgesia was lowered by two-fold when evaluated for heat sensitivity, and was strongly decreased for mechanical allodynia (Figure 6A), indicating that mu receptors on Nav1.8-neurons contribute to morphine anti-hyperalgesic properties. Using a pharmacological approach, we verified the peripheral action of systemic morphine on CFA-induced hypersensitivity under our experimental conditions. Naloxone methiodide, an opioid antagonist that does not cross the blood brain barrier, has been used previously to investigate peripheral opioid receptors involvement [4]. When administered to mu^fl^ mice prior to morphine, naloxone methiodide inhibited morphine-induced analgesia (Figure 6C). Fentanyl, another widely used mu opiate agonist, was also tested on cKO and mu^fl^ mice. Mu-cKO animals showed diminished fentanyl-induced antiallodynia at the low 0.03mg/kg dose for mechanical hypersensitivity (Figure 6B).

In order to assess the role of peripheral mu receptor at later time-points of inflammatory pain, we induced inflammation by CFA administration into the tail and heat sensitivity was measured until day 9 (Figure 7B). Mu-cKO mice and floxed controls show no difference in heat hypersensitivity at days 2, 6 and 9. Conditional KO animals had a decreased analgesic response to morphine at day 9, indicating an implication of the targeted receptors at this later time point, hence strengthening the results obtained at day 2 in the paw inflammation model.

**Figure 8:**
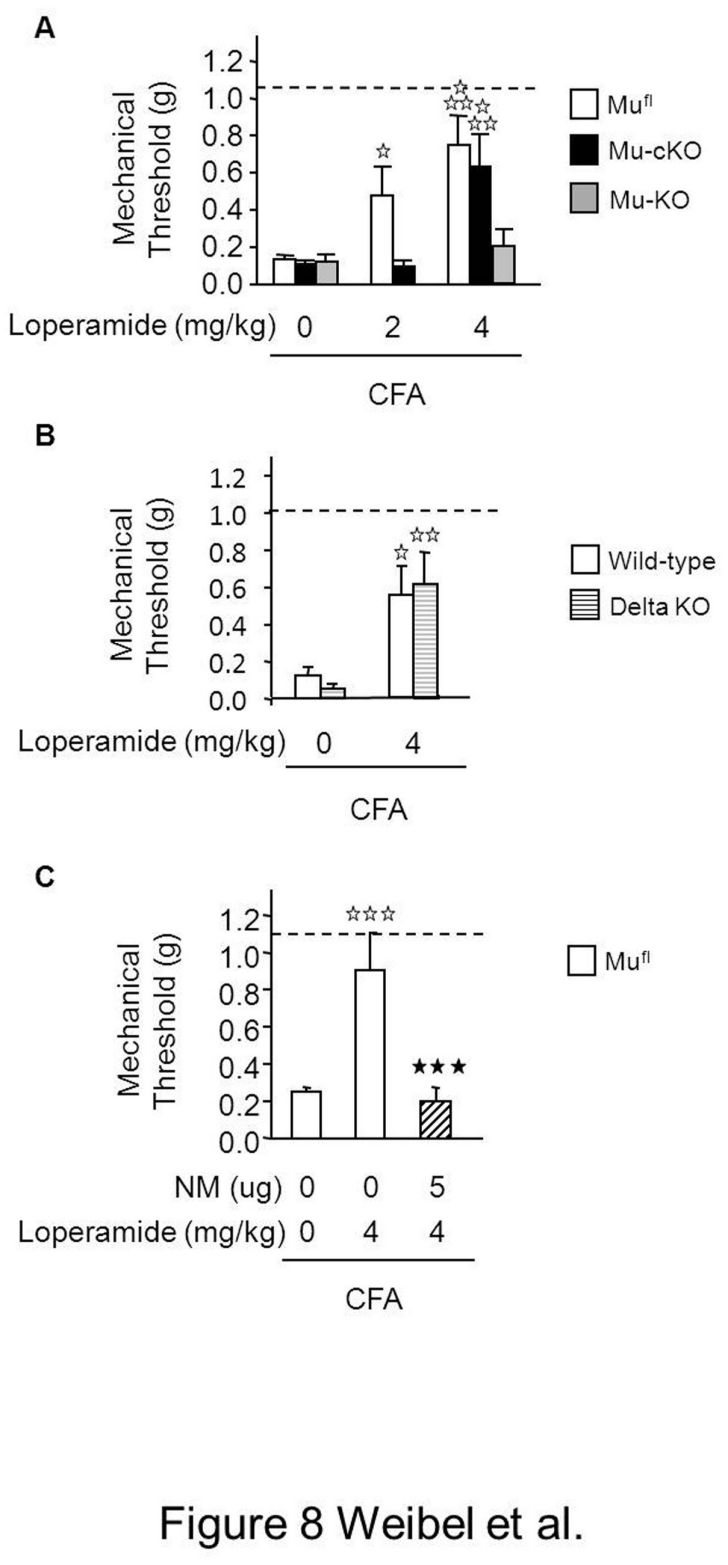
Conditional mu-cKO mice show decreased analgesia to the peripheral opiate loperamide in the paw inflammatory pain model. (**A**) Two-days after CFA, loperamide (s.c.) dose-dependently reduced mechanical hypersensitivity of mu^fl^ control mice. Analgesia produced by 2mg/kg loperamide was diminished in the conditional mutant mice and 4mg/kg loperamide induced no analgesia in the full mu-KO mice. Dashed lines represent baseline (pre-CFA) sensitivity. White bars, mu^fl^ mice; black bars, mu-cKO mice; grey bars mu-KO mice; n=4-10/genotype. Two-way ANOVA for mu^fl^ and mu-cKO mice (genotype X treatment F(1,78) = 17.508, *P* <0.001 for treatment; *P* = 0.0657 for genotype), post-hoc Bonferroni test, ✰ *P* <0.05, ✰✰✰ *P* <0.001, loperamide *vs* saline. (**B**) Loperamide-induced antihyperalgesia was comparable in control and delta opioid receptor total KO mice; n=9-13/genotype. White bars, control mice; stripped bars, delta receptor KO mice, two-way ANOVA (genotype X treatment F(1,38) = 16.363, *P* <0.001 for treatment; *P* = 0.959 for genotype), post-hoc Fisher test, ✰ *P* <0.05, ✰✰ *P* <0.01, loperamide *vs* saline. (**C**) Analgesia produced by 4 mg/kg loperamide in mu^fl^ mice was abolished by pretreatment with the peripheral antagonist naloxone methiodide (NM); n=11/group, white bars, mu^fl^ mice; hatched bars, NM-pretreated mu^fl^ mice; one-way ANOVA for treatment F(2,42) = 16.925, *P* <0.001 for treatment; post-hoc Bonferroni test , ✰✰✰ *P* <0.001, loperamide *vs* saline; ★★★ *P*<0.001, loperamide vs NM + loperamide.

Additionally we tested the analgesic potency of the peripherally acting mu opiate loperamide [32] in mu-cKO and control mice using the same paw inflammation model. Loperamide dose-dependently alleviated inflammatory hypersensitivity in mu^fl^ mice (Figure 8). In mu-cKO mice, 2mg/kg loperamide-induced analgesia was abolished, while analgesia was maintained at 4 mg/kg. At this 4mg/kg dose, loperamide-induced analgesia was absent in mu-KO mice (Figure 8A), confirming the selective action of loperamide on mu receptors. In order to determine if loperamide would also induce analgesia also via delta receptors, as shown by Shinoda and colleagues in a neuropathic pain model [33], we tested loperamide activity on delta receptor knockout mice. Loperamide-induced analgesia was maintained in delta-receptor KO animals (Figure 8B), demonstrating the selectivity of loperamide for the mu receptor. We then determined whether loperamide-induced analgesia at 4 mg/kg was due to the activation of central receptors or of peripheral mu receptors remaining in cKO mice, by pretreating floxed animals with the peripheral opioid antagonist naloxone methiodide. Naloxone methiodide pretreatment blocked the 4 mg/kg loperamide-induced analgesia (Figure 8C), indicating that remaining peripheral mu receptors likely expressed in non-Nav1.8-positive neurons, also contribute to the peripheral analgesia induced by loperamide.

### Mu opioid receptor expression is increased in DRGs from inflamed mu^fl^ control but not mu-cKO mice

**Figure 9:**
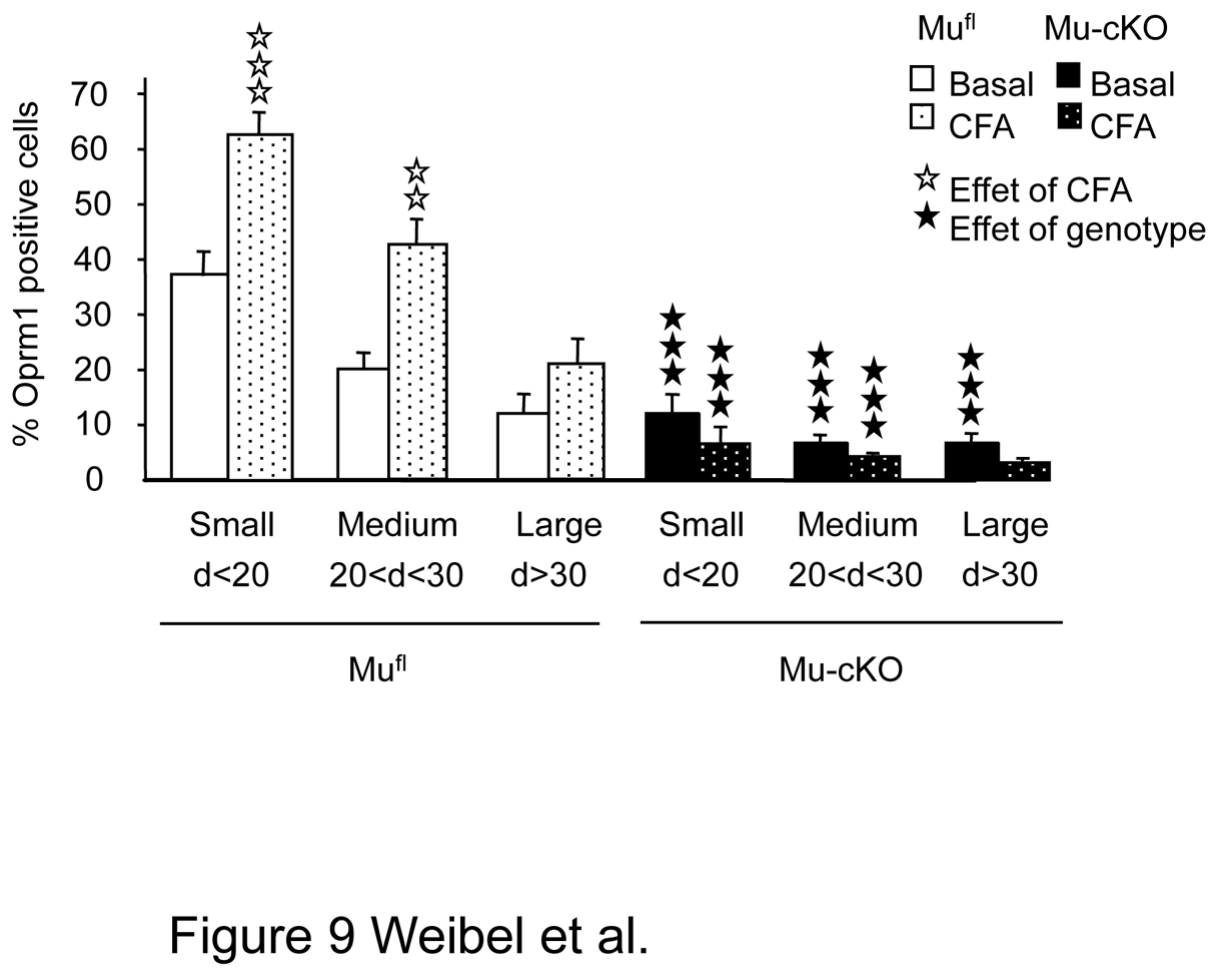
Inflammation increased the number of small/medium *Oprm1*-positive neurons in DRGs of mu^fl^ but not of mu-cKO mice. Inflammation was induced by intra-paw CFA as in previous figures. The cell size distribution of *Oprm1*-positive neurons in DRGs was evaluated by *In* *Situ* Hybridization. The % of *Oprm1*-positive neurons in naïve mu^fl^ and mu-cKO DRGs are shown in white and black, respectively. The % of *Oprm1*-positive neurons in ipsilateral DRGs of CFA mu^fl^ and mu-cKO DRGs are shown in dotted white and black bars. ✰✰ *P* <0.01, ✰✰✰ *P* <0.001, CFA *vs* naïve; ★★★ *P*<0.001 mu-cKO *vs* mu^fl^, Student t-test.

Our data indicate that peripheral mu receptors contribute to morphine analgesia in an inflamed, but not naïve state. Inflammation is known to increase expression of mu receptors in Nav1.8 neurons [4]. One explanation for our result could thus be that CFA indeed increases mu receptor expression in Nav1.8 neurons from mu^fl^ animals, to levels that allow significant morphine analgesia. In contrast, receptor levels would remain low in Nav1.8 neurons from mu-cKO animals, which would be less sensitive to peripheral morphine analgesia. In order to test this hypothesis, we evaluated the number of mu receptor-expressing neurons in the DRGs of both mu-cKO and mu^fl^ mice 48h after CFA, using ISH as above (Figure 1). Inflamed mu^fl^ mice showed a strong increase in the percentage of *Oprm1*-expressing neurons, in both small and medium size cells (Figure 9), in accordance with earlier findings [34]. In contrast, the percentage of *Oprm1*-expressing cells in DRGs from inflamed mu-cKO animals was unchanged after CFA. Therefore, morphine analgesia in mu^fl^ animals likely results from CFA-induced increased mu receptor expression in Nav1.8 neurons, which does not occur in the conditional mutants.

### Morphine-Induced Constipation Is Unchanged in mu-cKO Mice

**Figure 10:**
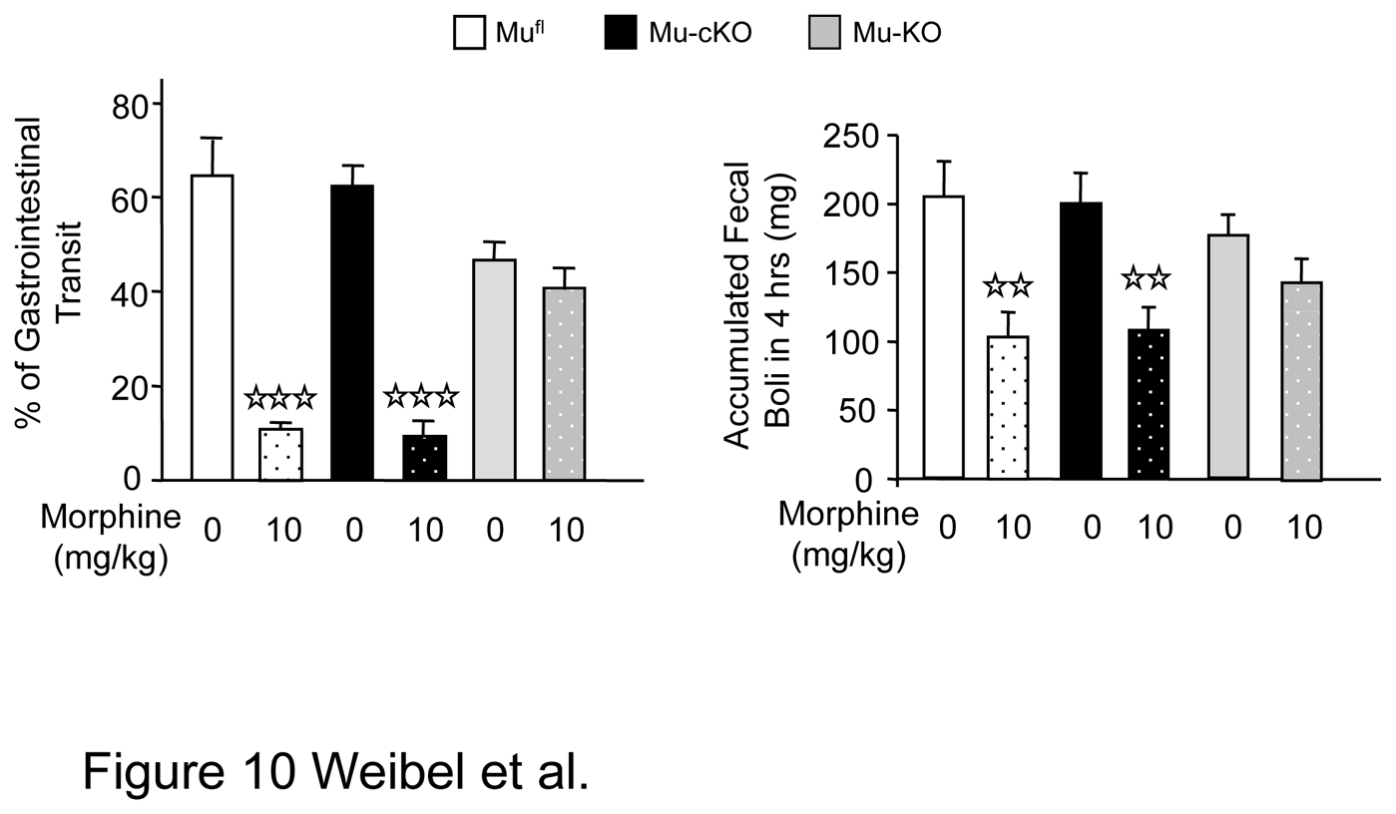
Morphine-induced constipation is maintained in conditional mu-cKO mice. Left: Morphine-induced inhibition of small intestinal transit. Mice were treated with saline (0 mg/kg morphine bars) or morphine (10 mg/kg) and 20 min. later given a charcoal gavage. For determination of small intestine transit, the distance travelled by charcoal was measured relative to the total length of the small intestine. White bars, mu^fl^ mice; black bars, mu-cKO mice; grey bars, mu-KO mice, n=5/group, two-way ANOVA (genotype X treatment F(2,24) = 18.206, *P* <0.001 for treatment; *P* = 0.184 for genotype), post-hoc Fisher test, ✰✰✰ *P* <0.001, morphine *vs* saline. Right: Morphine-induced inhibition of fecal boli accumulation. Mice were administered morphine or saline and fecal boli were collected after 4 hrs. n=10-12/group, two-way ANOVA (genotype X treatment F(2,62) = 21.138, *P* <0.001 for treatment; *P* = 0.934 for genotype), post-hoc Fisher test, ✰✰ *P* <0.01, morphine *vs* saline.

Genetic tracing experiments recently showed that some myenteric neurons express the Nav1.8-Cre protein [35]. In order to investigate any potential role of the targeted mu receptors in intestinal motility, we analyzed morphine-induced constipation by using small intestinal transit and fecal boli accumulation assays [25]. We observed a slight tendency to lower basal fecal boli accumulation in mu receptor total KO mice as compared to floxed mice (Figure 10A, two-way ANOVA for floxed and total KO mice, genotype X treatment F(1,16) = 1.541, *P* = 0.2323 for genotype). A slightly lower transit activity in conventional mu receptor KO mice has been described earlier [26], that was proposed to be due to compensation for the lack of mu receptor activity that normally decreases intestinal transit. In both tests, morphine-induced constipation occurred similarly in mu-cKO and mu^fl^ mice whereas morphine effect was abolished in mu-KO mice (Figure 10). These data show that mu receptors expressed by Nav1.8 neurons are not implicated in morphine constipating effects.

## Discussion

Both clinical and preclinical [4] studies have shown the potential for developing peripheral mu opioid analgesics to avoid central side effects. The implication of peripheral receptors has been investigated mainly based on pharmacological approaches and the use of peripheral antagonists [36-38]. In the present study, we reproduced these pharmacological data, and more importantly, our data provide the first genetic demonstration for the implication of mu receptors on primary afferent neurons in opiate-induced analgesia.

Mu opioid receptors are expressed throughout pain-controlling networks in the brain, in the spinal cord as well as in sensory neurons [2,11,39]. Our conditional knockout strategy in primary afferent Nav1.8 neurons produced a threefold decrease in mu receptor mRNA levels and mu receptor-expressing neurons in the DRGs where cell bodies for sensory neurons are located. The deletion of mu receptors occurs in small and medium diameter DRG neurons that represent the main population of primary nociceptive neurons including C fibers and A-delta fibers, while large diameter A-alpha/A-beta somatosensory neurons still express the mu receptor. These findings are in accordance with the well-described conditional deletion profile obtained with this Nav1.8-Cre mouse line [13,14].

Mu receptor cKO mice displayed pain sensitivity similar to control mice in all nociception assays investigated. Our results demonstrate that enhanced sensitivity to heat pain, which was previously observed in mu receptor conventional knockout or knockdown animals [29-31], implicates mu receptors expressed by cell types other than primary Nav1.8 neurons. These could be neurons of the central nervous system (brain or spinal cord) or Nav1.8-negative DRG neurons at the periphery. The present data are similar to those indicating no alteration of acute pain perception in animals with a conditional deletion of delta opioid receptors in these same neurons [15], and differ from those obtained with the Nav1.8-conditional deletion of cannabinoid CB1 receptors, that showed increased sensitivities to heat and pressure [40]. Overall endogenous cannabinoid but not opioid systems appear to regulate acute pain perception at the level of Nav1.8 sensory neurons. Our data also indicate that, under basal non-chronic pain conditions, and by using three different assays for heat pain implicating fast or delayed responses [41], one assay for mechanical pain and one measure of chemical pain, the targeted mu receptors on peripheral Nav1.8 neurons are not mainly implicated in the analgesic effects of opiates.

Further, in the CFA-induced inflammatory pain model, mu-cKO and control mu^fl^ animals developed comparable hypersensitivity, suggesting that mu receptors on Nav1.8 neurons are not principally involved in the tonic inhibition of CFA-induced inflammatory pain. Mechanisms implicating peripheral endogenous opioid tone for attenuating chronic pain were described previously [4,15,42]. In the future, Nav1.8 mu-cKO mice may be studied using other chronic pain models to examine whether peripheral mu receptors contribute to alleviate these chronic pain modalities such as neuropathies [38,43], diabetes [44], cancer [45,46] or cancer chemotherapy [47]. Also, peripheral mu and delta receptors may be both needed, or act synergistically, to produce a substantial endogenous opioid analgesia, as suggested by pharmacology studies [48-50].

In the several acute nociception assays tested, morphine-induced analgesia was similar in mu-cKO and control mice. This indicates that mu receptors expressed by primary Nav1.8 neurons are not predominantly implicated in morphine-induced antinociception. However, when morphine was tested in the CFA-inflammatory pain model, a significant part of the analgesia was lost in mu-cKO animals. Opiate analgesia was also substantially reduced in mu-cKO animals for low doses of fentanyl and loperamide, in accordance with recent pharmacological data suggesting an implication of peripheral mu receptor-mediated mechanisms for moderate, but not high doses of these opiates [51-53]. Analgesia data on inflamed mice from our genetic model, therefore, definitely establish that mu receptors on primary afferent Nav1.8 neurons represent key actors for systemic opiate analgesia under conditions of inflammatory pain.

Analgesia can also be obtained following intrathecal opiate administration that may be mediated by mu receptors on sensory neuron terminals in the spinal cord dorsal horn, spinal interneurons and projection neurons as well as neurons in the brain [54-56]. The study by Mansour and colleagues [2] has shown that spinal cord cells expressing mu receptor transcripts are localized in laminae 4 to 10, with fewer cells in laminae 2 and 3, and several other papers report a substantial mu receptor expression by spinal neurons by using RT-PCR [57-59]. Following dorsal rhizotomy, 40 to 72% [60-62] of mu receptor was lost in the spinal cord that was proposed to correspond to the loss of presynaptic receptors and possibly transsynaptic degenerative mechanisms. The analysis of presynaptic receptor involvement in opiate spinal analgesia has led to controversial results. In rats, paw inflammation and arthritis led to an increase in mu receptor expression in DRGs that did not translate into an augmented receptor level in spinal cord [63,64]. Also, paradoxically, the loss of Trpv1 primary afferent neurons obtained following resiniferatoxin treatment produced a reduction of spinal mu receptors but a potentiation of spinal opioid analgesia [65]. We found comparable mu receptor expression levels in the spinal cord of mu-cKO and control mice (Figure 2). This suggests that mu receptor expression by local spinal neurons could hide the primary afferent component, precluding the analysis of presynaptic receptor involvement by simple comparison of spinal analgesia in cKO and control animals. In the future, the role of mu receptors expressed by the remaining small/medium or large Nav1.8-negative DRG neurons as well as by neuronal populations in spinal cord and brain may be elucidated by the study of novel conditional KO mouse lines harboring a mu receptor deletion in these other specific neurons.

Here, the deletion of the mu receptor population potentially involved in primary nociceptive processing reveals the role of these receptors in systemic opiate analgesia under inflammatory condition but not in basal non-inflamed state. Previous research has shown that chronic pain or stress lead to enhanced opiate analgesia [36,66,67], potentially mediated by changes in mu receptor expression, localization or activity under these circumstances [34,63,64,68-70]. Accordingly, we found that CFA-induced inflammation increased the number of small and medium sized neurons expressing mu receptor in the DRGs of control mu^fl^ mice, a phenomenon that did not occur in the conditional mutants (Figure 9). These findings hence suggest that the augmented mu receptor expression in control animals during inflammation, which is absent in the conditional mutants, contributes to opiate-induced analgesia.

In conclusion, opiate-induced analgesia can be elicited by mu opioid receptor activation at several sites of the pain-control pathways within the nervous system and our approach identifies a specific mu opioid receptor population within sensory neurons as a key player for opiate-induced analgesia under inflammatory pain. It thus provides a strong basis for the design of peripherally acting opiate analgesics devoid of centrally mediated side effects [71]. Other mu receptor populations that contribute to pain control and opiate analgesia, in neurons other than Nav1.8 neurons and for distinct pain modalities, remain to be characterized by genetic approaches. To this respect, this mu receptor floxed mouse line represents a unique tool that, in principle, allows deletion of mu receptors at any specific sites or neuronal type of interest within pain pathways, using appropriate driver Cre transgenic mouse lines, or virally-mediated Cre excision.

Of broader interest, this mu receptor floxed mouse line constitutes an unique genetic tool that will be used to investigate the role of specific mu receptor populations in other pathologies of the nervous system including addiction or mood diseases [3,72].
